# Efficient and wavelength-tunable second-harmonic generation toward the green gap

**DOI:** 10.1126/sciadv.adw2781

**Published:** 2025-07-02

**Authors:** Zhiquan Yuan, Jinhao Ge, Peng Liu, Bohan Li, Mingxiao Li, Jin-Yu Liu, Yan Yu, Hao-Jing Chen, John Bowers, Kerry Vahala

**Affiliations:** ^1^ T. J. Watson Laboratory of Applied Physics, California Institute of Technology, Pasadena, CA 91125, USA.; ^2^ ECE Department, University of California, Santa Barbara, Santa Barbara, CA 93106, USA.

## Abstract

Achieving compact and efficient visible laser sources is crucial for a wide range of applications. However, traditional semiconductor laser technology faces difficulties in producing high-brightness green light, leaving a “green gap” in wavelength coverage. Second-harmonic generation (SHG) offers a promising alternative by converting near-infrared sources to visible wavelengths with high efficiency and spectral purity. Here, we demonstrate efficient and tunable SHG within the green spectrum using a high-*Q* Si_3_N_4_ microresonator. On-chip green power as high as 5.3 milliwatts is generated with a conversion efficiency of 141% per watt (absolute 7.9%). A space-charge grating induced by the photogalvanic effect realizes reconfigurable grating numbers and flexible wavelength tuning over a range of 2.6 terahertz. In addition, grating formation dynamics and competition are observed. These findings underscore the potential of Si_3_N_4_ as a robust, integrative platform for on-chip, tunable green light sources.

## Teaser

Tunable, efficient green light is generated by frequency doubling infrared light in a high-*Q* Si_3_N_4_ microresonator.

## INTRODUCTION

The development of compact, coherent visible laser sources is key to a range of applications in both science and engineering, including optical clocks (*1*), biomedical imaging (*2*), quantum information processing (*3*), and laser displays (*4*). However, it is challenging to create green light with high brightness and efficiency from traditional semiconductor laser technologies due to material limitations and low quantum efficiency (*5*). This issue is commonly referred to as the “green gap” (*6*), a wavelength span around 500 to 550 nm. Dye lasers based on organic molecules offer wide wavelength tunability. However, like other traditional bench-top solutions, they are bulky and complex, limiting their use in compact applications (*7*). Also, quantum dot lasers have high tunable range and are suitable for on-chip integration but still suffer from low efficiency and coherence at the green gap (*8*, *9*).

Nonlinear optical phenomena in high-*Q* resonators provide another approach for achieving new wavelengths (*10*). For instance, optical parametric oscillation (OPO) based on four-wave mixing (FMW) in microresonators (*11*, *12*) has been extensively studied in recent years (*13*–*15*) and enables access to the green gap (*16*). Alternatively, green light generation has been reported by third-harmonic generation (*17*–*19*) and cascaded sum frequency generation (*20*), but with limited efficiency. In contrast, second-harmonic generation (SHG) offers high conversion efficiency and coherence by frequency doubling from near-infrared (near-IR) sources (*21*–*27*). Among various photonic platforms for SHG, Si_3_N_4_ is particularly promising due to its ultralow optical loss, complementary metal-oxide semiconductor (CMOS) compatibility, and suitability for nonlinear optics. Recently, photogalvanic effect–induced second-order nonlinearity has been observed in Si_3_N_4_ with efficient SHG (*28*–*32*).

**Fig. 1.**
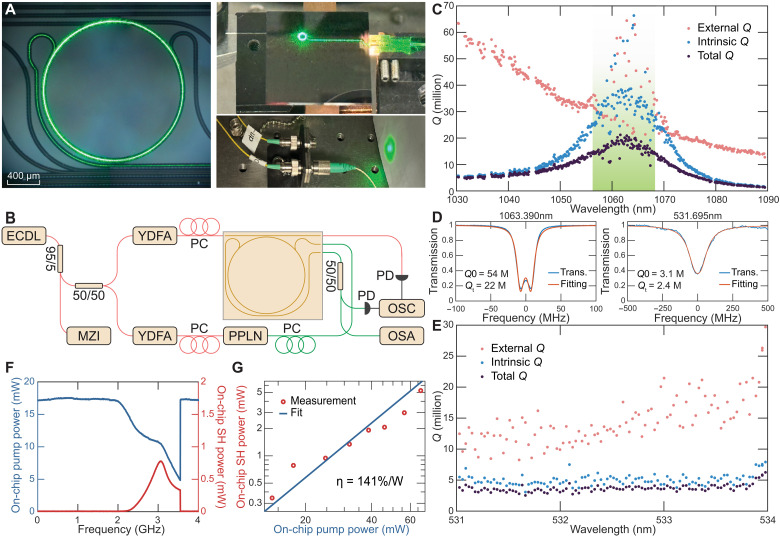
Device characterization and SHG performance of the Si_3_N_4_ microresonator. (**A**) Photographs of the ring resonator and off-chip green light emission during SHG operation. (**B**) Experimental setup for frequency matching tuning and SHG performance characterization of the Si_3_N_4_ microresonator. The chip temperature is controlled and stabilized by a thermoelectric cooler (not shown). Abbreviations: ECDL, external cavity diode lasers; MZI, Mach-Zehnder interferometer; YDFA, ytterbium-doped fiber amplifier; PPLN, periodically poled lithium niobate; PC, polarization controller; PD, photodetector; OSC, oscilloscope; OSA, optical spectrum analyzer. (**C**) Measured quality (*Q*) factor distribution versus wavelength from 1030 to 1090 nm, with the designed high-*Q* band highlighted in green. (**D**) Transmission spectrum and fitting of the pump resonance at 1064 and 532 nm. (**E**) Measured *Q* factor distribution versus the wavelength from 531 to 534 nm. (**F**) On-chip transmission of pump power (left axis) and SHG power (right axis) during pump laser frequency scanning across a cavity resonance at the phase-matching condition. (**G**) On-chip SHG power (red dots) versus pump power. The frequency conversion efficiency is fit by the blue line with a slope of 2, indicating a constant SHG efficiency (η, in %/W) within the measured range.

In this work, we leverage thin-film, ultralow-loss Si_3_N_4_ (*33*) to achieve efficient SHG in the green spectral range. By using a carefully designed coupler to minimize leakage loss, we demonstrate a broadband high-*Q* region and strong green emission with high coherence (see Fig. 1A). In the photogalvanic-induced quasi-phase-matching process, optically induced space-charge gratings are reconfigurable, enabling robust, dynamically tunable SHG in real time. Furthermore, we observe dynamic grating competition during space-charge formation. The tunability, combined with the maturity of Si_3_N_4_ as a platform for photonic integration, positions this system as an ideal candidate for versatile, on-chip green laser sources.

## RESULTS

### Device characterization

The device is fabricated based on an ultralow-loss Si_3_N_4_ platform (*33*, *34*). The Si_3_N_4_ resonator waveguide core is a 5-μm wide by 50-nm thick design with an upper cladding of 2.2-μm thick silica. The design has a radius of 850 μm, corresponding to a free spectral range (FSR) of 36.61 GHz at 1064 nm. To facilitate efficient coupling in both the near-IR and visible bands, the design incorporates two pulley couplers (see Fig. 1A) for spectral partitioning. The upper coupler, with a 3.5-μm gap, enables injection of the near-IR pump laser while minimizing coupling to the visible (green) mode, and the lower coupler, with a 0.3-μm gap, efficiently extracts the SHG signal while reducing the leakage of the near-IR mode.

The microresonator is characterized using the setup illustrated in Fig. 1B. A 1064-nm tunable laser is split into two beams. One beam serves as the probe wave for the fundamental near-IR modes and provides the pump power for SHG, whereas the other beam is amplified and frequency doubled by a periodically poled lithium niobate (PPLN) crystal to probe the green resonances. A four-channel V-groove array (VGA) and a lensed fiber are used for simultaneous coupling to both the near-IR and green waveguides.

The quality factor distribution in the near-IR band is first measured using a calibrated Mach-Zehnder interferometer (MZI) in combination with a wavelength-tunable laser (*35*). A main contribution to optical loss in the near-IR band is optical leakage through the visible coupler. Accordingly, the visible coupler was designed to minimize near-IR loss around the 1064-nm pumping wavelength. As shown in Fig. 1C, the measured *Q* distribution is maximal around this design target where, within a 12-nm bandwidth, the total *Q* factor exceeds 15 million (highlighted in green). The scatter in the *Q* data in this region is an artifact of measurement near the critical coupling point. In addition, we characterize the *Q* distribution of the green modes by scanning the pump frequency while simultaneously tuning the PPLN crystal temperature to maintain sufficient SHG probe power (Fig. 1E). Within the measurement range, the green modes are undercoupled and their total *Q* factors are clustered around 3 million, which shows the low absorption loss of the material (*36*, *37*).

In the photogalvanic process, a space-charge grating creates a field that works with the existing Kerr effect to create an effective second-order nonlinearity. The grating also ensures quasi-phase-matching by compensating for the phase difference between fundamental and SHG fields (*31*). Therefore, only the frequency matching condition is necessary, where the frequency of the visible mode is required to be twice of the fundamental field. To accomplish this, chip temperature is tuned to align green and near-IR modes. During this process, the green resonance is monitored by PPLN doubling of the pump laser. It is worth noting that, even without an external green probe signal, the near-IR pump light alone is sufficient to generate a weak seed signal, enabling the grating formation. The long-lived electron traps ensure that the space-charge grating remains stable over multiple days without decrease in SHG efficiency (*29*).

At a chip temperature of 25.0°C, a pump resonance (1063.390 nm) aligns with a visible resonance (531.695 nm), and photogalvanic-induced SHG occurs automatically. The *Q* factors of the pump and SHG modes are shown in Fig. 1D. When the 17-mW input pump laser is launched into the waveguide and scanned across the near-IR resonance, SHG power as high as 0.78 mW is generated on chip, corresponding to an efficiency of 250%/W (Fig. 1F). For the same resonance, the on-chip SHG power is also measured versus pump power in Fig. 1G, with a conversion efficiency of 141%/W averaged over input power levels. The SHG power reaches 5.3 mW in the bus waveguide at the input power of 67 mW (absolute efficiency of 7.9%). The green probe signal is turned off during this measurement. On the basis of the measured SHG efficiency and quality factors, the effective $\chi^{\left(2\right)}$ is estimated to be 0.022 pm/V. However, we note that the calculation of effective $\chi^{\left(2\right)}$ involves multiple assumptions, and certain parameters may vary. As a result, there are substantial uncertainties in this estimate.

### Wavelength tunability

**Fig. 2.**
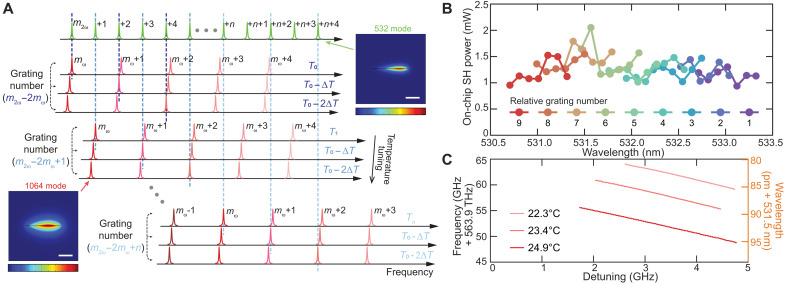
Coarse and fine wavelength tuning in green SHG. (**A**) Illustration of the multiple frequency matching condition in the SHG process for coarse tuning control. The first row shows multiple longitudinal modes of the fundamental transverse electric (TE) mode family in the green spectral region. Subsequent rows display the longitudinal modes of the fundamental TE mode at 1.06 μm, with different SHG frequency matching conditions (dashed lines) at the corresponding cavity temperatures. Within each group, the grating number ( $m_{\text{green}}-2m_{\text{near}-\text{IR}}$ ) of the photogalvanic space-charge field remains constant. Inset: Cross-sectional view of simulated mode profiles at the green SHG mode and near-IR pump mode, respectively. Scale bars, 2 μm. (**B**) Experimental measurements of the generated SHG wavelengths at different matching conditions. The *y* axis represents the on-chip SHG power, and the colors indicate the grating number difference between the pump and SHG modes. (**C**) Measured output green light frequencies are plotted versus the laser-cavity detuning at three different cavity temperatures for the same pump-SHG mode pair. The on-chip pump power is 78 mW. Continuous tuning is available at each point in (B), and this panel shows a representative example.

Concerning tuning of the emission, suppose at chip temperature $T_{0}$ , a one-to-one frequency matching condition is satisfied, where an SHG mode $m_{2\omega }$ is frequency matched with the pump mode $m_{\omega }$ , $f\left(m_{2\omega }\right)=2f\left(m_{\omega }\right)$ (as shown in Fig. 2A). Because the green and near-IR modes have different local FSRs and resonant frequency tuning coefficients ( δf/δT ) near these two modes, the frequency mismatch between the next pair of modes ( $m_{2\omega }+2$ and $m_{\omega }+1$ ) can be compensated through a reduction in chip temperature by $\Delta T=\frac{2\text{FSR}\left(m_{2\omega }\right)-2\text{FSR}\left(m_{\omega }\right)}{\mathrm{\delta f}/\mathrm{\delta T}\left(m_{2\omega }\right)-2\mathrm{\delta f}/\mathrm{\delta T}\left(m_{\omega }\right)}$ . This process can be cascaded to achieve multiple frequency-matched output wavelengths, with a constant optical poling–induced grating number ( $m_{\text{green}}-2m_{\text{near}-\text{IR}}$ ). The tuning mechanism is illustrated by multiple frequency matching conditions within a group of the same grating number (Fig. 2A), with each group corresponding to a single color in Fig. 2B. Tuning range is limited by the chip temperature range, which is kept between 17° and 60°C, allowing a 0.6-nm wavelength change in the green by steps of 0.1 nm.

Unlike noncentrosymmetric materials poled by high-voltage electrical pulses, where the resonator grating number is fixed during fabrication, the photogalvanic process allows for reconfigurable grating structures due to optically induced space-charge distribution (*31*). Accordingly, and in contrast to the above tuning process, the pump mode $m_{\omega }$ can be matched to adjacent green modes (temperature tuning), such as $m_{2\omega }+1$ (second group in Fig. 2A) by adjusting the temperature. In this case, $T_{1}-T_{0}=\frac{\text{FSR}\left(m_{2\omega }\right)}{2\mathrm{\delta f}/\mathrm{\delta T}\left(m_{\omega }\right)-\mathrm{\delta f}/\mathrm{\delta T}\left(m_{2\omega }\right)}$ . This process enables matching a single pump mode to multiple green modes, creating new colors in Fig. 2B. All experimentally measured on-chip SHG powers at different frequency matching conditions are plotted versus wavelength and categorized by the relative grating number. The optical spectrum analyzer (OSA) spectrum of each generated green signal is used to determine the SHG wavelength and on-chip SHG power, and the on-chip pump power is maintained at 39 mW. At the same chip temperature, increasing the relative grating number shifts the matching wavelength toward shorter wavelengths. The wide high-*Q* distribution at both wavelengths enables a maximum tunability of 2.6 nm (2.6 THz) with nearly constant SHG efficiency and minimum on-chip SHG power around 1 mW. There is no fundamental limit to the SHG wavelength tuning range, and the only restriction comes from the PPLN crystal used to probe the mode resonance frequencies.

Last, we also demonstrate the continuous fine tuning of the SHG wavelength by adjusting the pump laser frequency (laser-cavity detuning) within a single matching point in Fig. 2B. Because of the OSA’s resolution limit, a wavemeter (HighFinesse GmbH, WS6-600) is used to record the green light with 0.1-pm resolution. As shown in Fig. 2C, at 24.9°C, the SHG signal emerges when the laser-cavity detuning reaches 1.8 GHz. The SHG frequency then decreases at twice the rate of the detuning as it continues to increase, with the laser sweeping out of the cavity mode when the detuning reaches 4.8 GHz. When the chip temperature is slightly adjusted while maintaining the frequency matching condition, the cavity resonances also shift, thereby shifting the green light wavelength. By selecting three cavity temperatures, the output frequency can be continuously tuned by 16 GHz, which means each data point in Fig. 2B can occupy a width of 16 pm.

### Vernier matching and grating competition

**Fig. 3.**
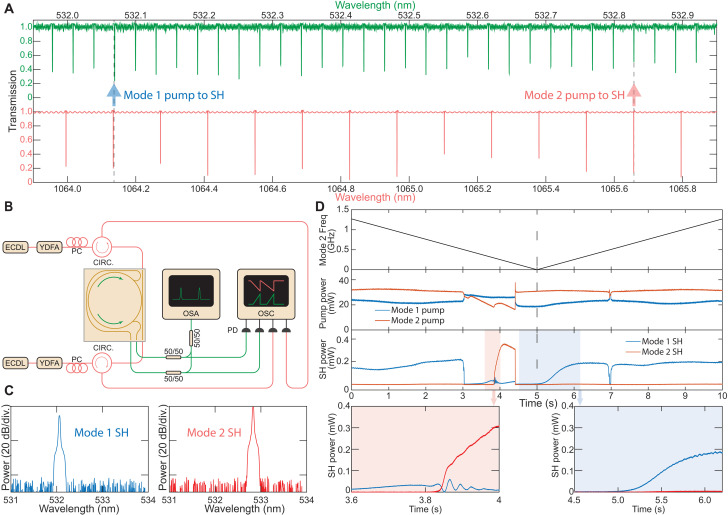
Vernier matching between green and near-IR modes and observation of grating competition dynamics. (**A**) Simultaneously measured frequencies of the mode families in green (green) and near-IR (red) bands are plotted versus wavelength at a chip temperature of 43.8°C. Vernier frequency matching is observed due to spectrally local FSR differences. Red and blue arrows indicate the positions of two distinct modes used for SHG. (**B**) Experimental setup for counterpumped SHG at two distinct wavelengths [mode 1 and mode 2 in (A)]. (**C**) Optical spectrum of the independently generated green light from two distinct pairs of modes. (**D**) Observation of the grating competition dynamics between two pairs of modes with different space charge grating numbers. The pump frequency of mode 2 is scanned periodically, whereas the pump frequency of mode 1 is unchanged and in resonance. Top trace: The frequency change of the mode 2 pump laser during scanning; middle trace: the transmitted on-chip pump power during scanning; bottom trace: the corresponding generated on-chip SHG power. Bottom insets: Zoom-in views of the corresponding shaded regions of the bottom trace, showing the grating built-up and erasure process.

Because of different local FSRs of modes at 532 nm (32.15 GHz) and 1064 nm (36.61 GHz), a Vernier frequency matching condition is also available. This allows alignment of mode pairs with different grating numbers at the same temperature. Following the method used in Fig. 1 (C and E), we measured the frequency dispersion of the modes in both the green and near-IR spectral bands simultaneously (shown in Fig. 3A). At a resonator temperature of 43.8°C, two distinct SHG alignments are observed at 1064.135/532.068 nm and 1065.658/532.829 nm. These are indicated by the blue (mode 1) and red (mode 2) arrows, respectively. The aligned modes are separated by 25 (11) modes in the green (near-IR) band, consistent with the FSR values previously measured (32.15/36.61 ≈ 22/25).

To distinguish the green light generated by each of the two modes, a counterpropagating (CP) pump setup is used (Fig. 3B). Here, two pump lasers are amplified and directed through circulators, generating two on-chip CP SHG signals. These signals are detected independently using photodetectors connected to the two output ports of the VGA. When a single pump laser is coupled into the resonator, SHG occurs automatically upon adjusting the pump wavelengths to match both resonances. The output green light spectra are shown in Fig. 3C. At this specific temperature, mode 2 achieves perfect frequency matching, whereas mode 1 has a slight residual misalignment (see Fig. 3A), resulting in a higher output power for mode 2.

The SHG process arises from photogalvanic-induced grating poling, where the SHG signal follows the formation of the corresponding grating in the microresonator. However, because these two green light signals originate from the same transverse mode family but differ in grating numbers, they produce unique electron distributions within the material. This introduces grating competition during the formation process of each grating.

To investigate the dynamics of this grating competition, we apply a 0.1-Hz ramp signal to gradually scan the pump laser frequency relative to mode 2 while keeping the mode 1 pump laser in resonance and fixed. In the first 5 s, the mode 2 pump laser frequency gradually shifts from blue to red, scanning from outside of mode 2, entering its resonance, and then moving out again. During the next 5 s, it reverses direction, scanning from red to blue across the mode 2 resonance. This frequency scan path is depicted in the top trace of Fig. 3D, with the corresponding transmitted pump power and SHG power on-chip displayed in the middle and bottom traces. At the beginning, the mode 1 pump laser is tuned into the mode 1 resonance, establishing the grating structure for mode 1 and enabling SHG. As the mode 2 pump frequency gradually scans into resonance, the SHG power from mode 2 increases whereas the SHG power from mode 1 decreases (zoom-in in the left inset). This observation indicates the formation of the grating for mode 2 and the erasure of the mode 1 grating, leading to a brief overlap where both green lights coexist. Fluctuations in mode 1 SHG power are attributed to interference induced by backscattering from mode 2. The grating formation for mode 2 occurs within a second, consistent with previous observations (*32*). Notably, the abrupt drop in mode 1 SHG power at *t* = 3 s results from a sudden loss of pump power due to thermal competition, whereas the gradual decrease after *t* = 3.8 s indicates grating reformation, accompanied by an increase in mode 2 SHG power. This highlights the contrast in timescales between two processes: Grating formation develops over seconds, whereas thermal effects manifest within milliseconds.

When the mode 2 pump laser scans out of resonance, it no longer influences the grating structure, allowing the mode 1 pump laser to reestablish resonance and rebuild its grating. Because the grating for mode 2 has already been formed, mode 1 SHG power does not regenerate immediately but instead increases gradually as the mode 1 grating is rebuilt and mode 2’s grating neutralizes (right inset of Fig. 3D). The observed relaxation time between the formation of the two gratings reflects the timescale associated with dynamic switching between modes. Mode 1’s grating takes longer to regenerate due to the slight mode misalignment.

For comparison, at *t* = 7 s, mode 1 SHG power drops as the mode 2 pump laser scans into resonance from the red side and change the cavity temperature. However, this brief resonance does not last long enough to create a new grating or fully erase the existing one, so mode 1 SHG power quickly returns after the mode 2 laser exits resonance.

## DISCUSSION

In summary, we have demonstrated efficient and tunable SHG in Si_3_N_4_ microresonators within the green spectral range. By leveraging photogalvanic-induced quasi-phase-matching, robust SHG was achieved with precise control over multiple frequency-matched modes. Table 1 summarizes the key metrics of this work and previous works for green light generation in integrated platforms. The high quality factors for both green and near-IR modes highlight the potential of Si_3_N_4_ microresonators for high-efficiency, stable performance across different frequency bands. The observed grating competition between SHG modes further reveals the dynamic behavior of photonic grating formation, suggesting avenues for more advanced control, including dynamic mode switching.

**Table 1.** Comparison of state-of-the-art green light generation in integrated platforms.

Work	Process	Material	Tunability	$\lambda_{\text{Pump}}$	$\lambda_{\text{Green}}$	Pump power	Green power	Normalized efficiency	Max absolute efficiency
This work	SHG	Si_3_N_4_	2.6 THz	1064 nm	532 nm	67 mW	5.3 mW	141%/W	7.90%
Ref. (*16*)	OPO	Si_3_N_4_	70 THz	766.2–778.8 nm	535–612 nm	50 mW	100–500 μW	20%/W	1%
Ref. (*19*)	THG	AlN/Si_3_N_4_	Not shown	1542 nm	514 nm	30 mW	49 μW	180%/W^2^	0.16%
Ref. (*20*)	Cascaded SHG+SFG	Lithium niobate	Not shown	1560 nm	520 nm	54 mW	334 μW	12,000%/W^2^	0.60%
Ref. (*26*)	Cascaded SHG+SFG	Lithium niobate	Not shown	1550 nm	517 nm	20 mW	2 μW	152% W^−2^ cm^−2^	0.01%
Ref. (*27*)	SHG	Lithium tantalate	0.2 THz	1064 nm	532 nm	16 mW	1.87 mW	1290% W^−1^ cm^−2^	11.7%

The results presented here offer several key advantages, including a wide transparency window, ultrahigh quality factors, compatibility with CMOS photonic integration, and excellent power handling capabilities. Si_3_N_4_ has demonstrated impressive capability for broader photonic integration, including heterogeneous and hybrid integration with active components such as III-V semiconductor lasers (*38*–*44*). Commercially available distributed Bragg reflector (DBR) laser diodes operating in the 1-μm band can deliver over 200 mW of output power. If hybridly integrated with the device demonstrated in this work, these lasers are expected to generate more than 10 mW of on-chip green light. Toward practical applications, maintaining long-term performance necessitates precise thermal management and optimized mechanical coupling to ensure stability and efficiency. In addition, the all-optical poling method does not require lithographically defined electrodes and subsequent poling with high-voltage electrical pulses. On the other hand, traditional $\chi^{\left(2\right)}$ materials, such as PPLN, provide larger $\chi^{\left(2\right)}$ nonlinearity, making them ideal for applications requiring efficient SHG at low input power levels.

Nonetheless, the achievable power in Si_3_N_4_ SHG can be notably enhanced by using higher-order modes for perfect phase matching (*30*) and through further boost in optical *Q* factors. Also, broadband SHG can be achieved through careful resonator design (*45*). With their high efficiency and photonic integration compatibility, this work shows that Si_3_N_4_ microresonators are promising candidates for on-chip tunable coherent green sources.
